# Leadership, capability and performance: A study among private higher education institutions in Indonesia

**DOI:** 10.1016/j.heliyon.2023.e13026

**Published:** 2023-01-18

**Authors:** Marvello Yang, Abdullah Al Mamun, Anas A. Salameh

**Affiliations:** a Institute of Technology and Business Sabda Setia Pontianak, Kota Pontianak, Kalimantan Barat, 78121, Indonesia; b UKM–Graduate School of Business, Universiti Kebangsaan Malaysia, 43600, UKM Bangi, Selangor Darul Ehsan, Malaysia; c College of Business Administration, Prince Sattam Bin Abdulaziz University, Al-Kharj, 11942, Saudi Arabia

**Keywords:** Organizational leadership capability, Learning orientation, Strategic flexibility, Ambidextrous capability, Organizational performance

## Abstract

Higher education institution is an important sector to obtain capabilities for global challenges. This study explored the mediating effect of ambidextrous capability between the link organizational leadership capability, learning orientation, and strategic flexibility on the performance of private higher education institutions in Indonesia. In this cross-sectional study, an online survey, which involved 524 rectors or deans in private higher education institutions (HEIs), was conducted using Google Forms. The accumulated data were analyzed using SEM-PLS 4.0. The findings revealed that organizational leadership capability displayed a statistically insignificant effect on organizational performance. However, learning orientation and strategic flexibility were found statistically significant effects on organizational performance. Moreover, this study also confirmed that organizational leadership capability, learning orientation, and strategic flexibility had a significant correlation with ambidextrous capability. The relationship between organizational performance and ambidextrous capability was identified as having no significant relationship. Apparently, organizational leadership capability, learning orientation, and strategic flexibility had no significant effect on organizational performance through ambidextrous capability. This study confirmed that ambidextrous capability had no mediating effect between organizational leadership capability, learning orientation, and strategic flexibility on the performance of private higher education institutions. This study found that dynamic capability theory (DCT) by identifying the mediating effect of the ambidextrous capability to gain organizational performance was not supported by the findings of this study. The ambidextrous capability may be applied to devise effective strategies and policies that can enhance organizational performance of different sectors.

## 1 Introduction

It has become increasingly competitive among higher education institutions (HEIs) when it comes to student enrolment, financial concerns, technology use, and diversified curriculum offerings [1]. Tabucanon et al. [2] identified curriculum programme as a critical issue for all higher education leaders to achieve sustainable performance. For instance, in order to support the performance of HEIs, the government of China provided financial support to ensure the quality of higher education [3]. Furthermore, the government of Indonesia supported the performance of HEIs in 2020 through the state budget allocation of 505.8 trillion rupiahs, with 1.463 trillion rupiahs for research and technology and 957.2 trillion rupiahs for other expenses [4]. Strategic engagement with capability concerns is required to support leaders and the success of HEIs. It is essential to integrate the capability, strategy, and curriculum in HEIs in order to identify opportunities and obstacles for the performance of HEIs [5].

HEIs have encountered various barriers in devising a proper strategy via external adaptation and internal integration to obtain sustainable organisational performance [6]. Apart from being a crucial factor to produce a professional workforce that can effectively eradicate poverty and boost economic progress in developing countries, the role of organisational leadership capability within the higher education domain is critical [7]. In addition, improving organisational leadership capability is necessary to obtain specific opportunities and adopt appropriate strategies for sustainable performance [8]. The majority of HEIs have not applied organisational leadership capability to achieve higher performance [9]. The role of organisational leadership capability in promoting organisational performance is indeed a well-studied field of study, with significant implications for HEIs [10].

Jansen et al. [11] defined ambidextrous capability as the capabilities to excel at using current resources for exploitative innovation and to develop new opportunities that can improve exploratory innovation simultaneously. Meanwhile, Tabucanon et al. [2] identified ambidexterity as a strategic focus that enhances organisational leadership capability through the development of capabilities that allow strategic combinations of exploration and exploitation to realise high-quality curriculum, student creativity, and knowledge in HEIs. Organisational ambidexterity involves the use of existing resources, capabilities, and responsibilities, while simultaneously exploring new resources, new capabilities, and new responsibilities. Therefore, ambidextrous capability is a crucial part of organisational strategies for HEIs. There is still a need to document the wide range of organisational leadership capability in relation to ambidextrous capability in HEIs, particularly in low- and middle-income nations [12]. There are limited studies on organisational leadership capability and ambidextrous capability, which was addressed in the current study. Besides that, it was deemed noteworthy for this study to explore the key determinants for the performance of HEIs, particularly in Asian countries.

This study concurred with the theoretical approach of dynamic capabilities, which contends that strategy and capability processes have embodied effects [12]. Dynamic capabilities gain other sources to attain strategic advancement in HEIs [13]. Successfully constructing strong dynamic capabilities allows organisations to prioritise efficiency over innovation [14]. A leader's strong dynamic capabilities can encourage high performance through new product development and reconfigured organisational capabilities and technological opportunities [15]. Wu et al. [16] claimed that dynamic capabilities encourage ambidextrous capability that exploits both internal and external capabilities to gain organisational sustainability.

The essential factors to obtain the performance of higher education institutions can be achieved by adopting Dynamic capability (DC). The study aims to explore the mediating effect of ambidextrous capability between the association organisational leadership capability, learning orientation, and strategic flexibility on the performance of higher education institutions. This study adopted dynamic capability theory to identify the strategy and capabilities. It is used to undergird organizational level sensing, seizing, and reconfiguring capacities that are difficult to develop and deploy. This study will also contribute to present the body of knowledge of the leadership literature in education through extending dynamic capability theory. Also, this present study provides practical and policy implication to obtain the sustainable performance of HEIs.

## 2 Literature review

### 2.1 Theoretical foundation

Using the dynamic capability theory driven by resource-based view (RBV), this study aimed to evaluate the mediating effect of ambidextrous capability on the relationships of organisational leadership, learning orientation, and strategic flexibility with organisational performance. Human resource and managerial resources are important asset for organization to create sustainable competitive advantage [17]. Thus, proper strategic implementations by increasing organisational capability, dynamic capability, organisational attribute, information, and knowledge are crucial to gain competitive advantages [18,19]. Dynamic capabilities help organisations to understand their collective knowledge and these capabilities (intangible assets) become crucial assets due to the difficulty in imitating them by competitors. Based on the dynamic capability theory, global competitive environments, consumer needs, technological opportunities, and competitor activity are constantly in a state of flux [20,21].

### 2.2 Dynamic capability approach

As Teece [22] described, dynamic capabilities are an organisation's capability to change the internal and external competencies into strengths in the fast and unpredictable market. Dynamic capabilities are a vital key to face challenges and reconfigure threats and weaknesses to become competitive resources. Since higher education institutions respond slowly to changes, Hayter and Cahoy [23] have highlighted that organisation capabilities enable higher education institutions to expand their facilities, programmes, and services to generate revenue to gain better financial and non-financial performance. Various capabilities are embedded in routine and operational activities which are termed ordinary capabilities by Teece [22]. Ordinary capabilities determine how an organisation operationally performs short term sustainability practices and these capabilities are insufficient to achieve organisational success and survival in a competitive environment, especially in developing countries [22]. Nonetheless, dynamic capabilities enable an organisation to change, sense, and seize new business opportunities and then utilise these opportunities in new value-creating strategies by modifying the ordinary capabilities.

Ambidextrous capability is defined as an organisation's capability to transcend at utilising available resources to form exploitative innovation, encourage creativity and develop new opportunities to collectively improve explorative innovation [11]. Innovation can be assessed using organisational behaviour and mindset to balance the correlation between the capability of exploitative and explorative to achieve sustainability. The ambidextrous capability theory has a significant role in strategies by adopting dynamic capability. Zeng et al. [24] have emphasised how ambidextrous capability generates the differentiation and integration of capabilities that form individuals' behaviour to attain sustainable performance. The differentiation and integration are considered the strategy, method, and drive to achieve innovation. Ambidextrous capability represents seizing opportunities in terms of dynamic capability through the reconfiguration, combination, and transformation of existing knowledge and new resources such as technology or market [25,26].

On the other hand, ambidextrous leadership is termed as the integration of an authoritative and imperative leadership style [27]. An empowering leadership provides workers with the authority to effectively support their responsibility and decision making regarding their work and resources. Ambidextrous leadership with an empowering and directive leadership style boosts or reduces stress besides easing the restrictions affecting the subordinates’ motivation through an appropriate leadership style [28]. The different leadership capabilities warrant organisations to compensate the competing constraints of the various environments in the unpredictable market to perform effectively which in Chinese is termed the Yin-Yang balance.

Moreover, the learning process is a critical process of ambidexterity to obtain knowledge for suitable decision making and problem-solving in rapidly changing environments by integrating prior knowledge and exploring new knowledge [29]. The responsibility and challenge of organisational ambidexterity are dependent on internal and external factors. Kivipold [30] has stated that leadership competence is a combination of knowledge and skills to form effective leadership. Furthermore, an organisation needs to continuously improve its technological resources and capabilities to be better equipped to gain distinctive advantages instead of transient advantages. Therefore, this study applies the dynamic capability theory (DCT) to explain the role of ambidextrous capability to obtain sustainable organisational performance.

### 2.3 Hypothesis development

#### 2.3.1 Organizational leadership capability

As a leader in an organization, leadership capability is needed to improve work attitude and performance through innovation and mindset transformation [31]. Innovation and mindset transformation are regarded as attributes of dynamic capability to achieve new idea and attitude, which are promoted by leadership capability [32]. In addition, organisational leadership need to seize opportunity and innovative strategy in order to improve short- and long-term sustainable organisational performance [33]. Leadership of HEIs must interact with ambidexterity in order to resolve innovation and creativity dilemmas for sustained competitive advantage [34]. Hence, based on the literatures above, the following hypothesis is proposed.

Hypothesis 1a (H1a): Organizational leadership capability has a positive effect on ambidextrous capability among private higher educations.

Hypothesis 1b (H1b): Organizational leadership capability has a positive effect on organization performance among private higher educations.

#### 2.3.2 Learning orientation

In RBV, knowledge is a basic core of competency, especially for capability innovation from investment in knowledge management [35]. The awareness of new knowledge on consumer's needs in many platforms is considered as a priority in strategic formulation. The knowledge transfer in strategy is crucial to create new value positions to obtain innovation by exploring consumer's purchasing experience [36]. Learning orientation is important attribute in strategy formulation which facilitates the accumulation of past experience of organization into new knowledge or the capability in creating new innovation through exploitation and exploration [37]. **The capability to integrate, build, and reconfigure internal and external competency is needed as an implementation of strategy** [21]**.** Moreover, Yang et al. [38] stated that interactive learning orientation between consumer's experience and interpretation of organization enhances innovation by knowledge acquisition. Furthermore, learning orientation enables strategy formulation to predict consumers' need [36]. Therefore, based on the previous studies above, the following hypothesis is proposed.

Hypothesis 2a (H2a): Learning orientation has a positive effect on ambidextrous capability among private higher educations.

Hypothesis 2b (H2b): Learning orientation has a positive effect on organizational performance among private higher educations.

#### 2.3.3 Strategic flexibility

From a resource-based perspective, flexibility is determined by the nature and adaptability of organizational resources and allocation of managerial attention. Through the balancing of adaptability and alignment oriented-decision, the decision-maker in organization must formulate the strategy to encourage ambidextrous innovation. The strategy relies on the target market that requires a nuanced appreciation of the link between structural change and managerial attention [39]. An organizational capability to adapt quickly is crucial to gain a success and thus, strategic flexibility enables an organization to obtain competitive advantage [40]. The focus of exploitation in current environmental conditions by adapting existing technologies requires organizational leadership to be more flexible in strategy to define consumer's needs. In addition, exploration capability requires risk-taking, experimentation, flexibility, and new information discovery to gain organization performance. Therefore, exploration and exploitation innovation require a different set of organization structure and processes to obtain performance [41]. According to the previous studies above, the following hypothesis is proposed.

Hypothesis 3a (H3a): Strategic flexibility has a positive effect on ambidextrous capability among private higher educations.

Hypothesis 3b (H3b): Strategic flexibility has a positive effect on organizational performance among private higher educations.

#### 2.3.4 Ambidextrous capability

Knowledge improvement, external analysing capability, and innovation are essential factors to understand consumers' need [42]. Recognising the opportunity, managing uncertainty, acquiring resource, and developing new service or products may contribute to a stronger competitive advantage [26]. Ambidextrous capability is an important source for innovation, organisational learning, and organisational improvement [13]. Ambidexterity is an organizational capability or the managerial capability to conduct and balance exploitation and exploration innovation. Furthermore, organizational capability may generate different mindset in building and maintaining the strategic decision-making, monitor organizational strategy towards organizational performance. Organisational capability may involve reorganising the organisation's focus on the goals and its leader's decision to develop exploitative and explorative assets [13,43]. Based on the previous studies above, the following hypothesis is proposed.

Hypothesis 4 (H4): Ambidextrous capability positively and significantly influences organisational performance among private higher education.

#### 2.3.5 The mediating effect of the ambidextrous capability

The organizational leadership capability is needed to adjust strategy formulation, and resource allocation during problem-solving and decision making to assure the managerial skill to create sustainable competitive advantage [39]. The capability to reformulate and obtain problem-solving skills needs an accurate data and information about consumers’ behavior which in turn to enhance organizational performance [43]. A leader in organization must reconfigure resource and the capability to identify external conditions in the emerging market [36]. Some studies revealed that ambidextrous capability plays mediating effect on organizational performance for different concepts [44]. Moreover, the reason for examining the mediating effect of ambidextrous capability becomes one of attributes of dynamic capabilities which are gradually developed over time to gain competitiveness [26]. Based on the previous studies above, the following hypotheses are proposed.

Hypothesis 5a (H5a): Ambidextrous capability mediates the effect of organizational leadership capability on organizational performance among private higher educations.

Hypothesis 5b (H5b): Ambidextrous capability mediates the effect of learning orientation on organizational performance among private higher educations.

Hypothesis 5c (H5c): Ambidextrous capability mediates the effect of strategic flexibility on organizational performance among private higher educations.

**Fig. 1:**
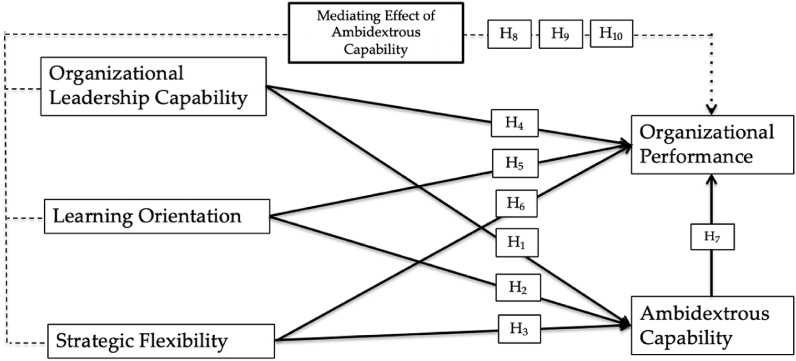
Research model.

Fig. 1 illustrates all of the hypothesized and tested associations in this study.

## 3 Methodology

This cross-sectional study targeted rectors and deans of selected private HEIs in Indonesia to evaluate the influence of organisational leadership capability, learning orientation, and strategic flexibility on organisational performance via ambidextrous capability. Structural equation modelling (SEM) was performed to test all hypothesized relationships (Fig. 1). In particular, this study examined the strength among latent variables and indicators using SEM-PLS 4.0. The Ministry of Education, Culture, Research, and Technology [45] recorded a total of 3115 HEIs in Indonesia.

### 3.1 Sampling

The required sample size for this study was calculated using G-Power software. With the power of 0.95 (greater than 0.80 as a requirement in social and behavioural science research) and the effect size of 0.15, this study required a sample size of at least 107 to analyse the model with six constructs. Private HEIs in Indonesia were targeted in this study because, unlike public HEIs, private HEIs have to create strategies to gain competitive advantage in terms of intensifying the capabilities of lecturers and the curriculum of the institution. Purposive sampling was used to select these private HEIs. A total of 1515 private HEIs were selected for this study based on the following criteria: (1) established for more than five years; (2) have more than 1000 students; (3) registered under the Ministry of Education, Culture, Research, and Technology of Indonesia. Following that, the rectors and deans of the selected 1515 private HEIs were invited to participate in the online survey via e-mail. After getting consent to participate in this survey, links to the online survey were sent to 700 private HEIs. All emails were sent to the respective human resource management offices of the selected private HEIs. The online survey was conducted from June 2022 to September 2022. Data from 524 rectors and deans of private HEIs in Indonesia were obtained for analysis. Around 74% of the participants in this survey responded.

A five-point Likert scale, with the endpoints of “strongly disagree” (1) and “strongly agree” (5) was used to measure the constructs. Through this Likert scale, respondents can specify their degree of agreement and disagreement with several assertions regarding attitude, object, perception, or event. A pilot test, which involved 30 colleges in Indonesia, was conducted prior to the actual survey. The results of pilot test confirmed the validity and reliability of all questionnaire items to measure the constructs.

### 3.2 Research instrument

The data were collected and analyzed to test the hypotheses by e-questionnaires. This study measured the organizational leadership capability by adopted from Kivipold et al. [30] with thirteen items. Learning orientation was adopted from Baker & Sinkula [46]; Wang [47]; Huang & Li [44] with fifteen items. Moreover, strategic flexibility was adopted by Roberts & Stockport [48]; Guo & Cao [49] with ten items, whereas ambidextrous capability was adopted from Jansen et al. [11] with eight items. To measure organizational performance, this study adopted from Kivipold et al. [30] with six items.

### 3.3 Common method bias

To reduce common method variance (CMB), respondents were informed that their replies would be reviewed anonymously and that there were no correct or incorrect responses [50]. This study utilized Harman's one-factor test to discover CMB, in which a single fixed factor extracted from all constructs explains less than 50% of the variation. The common method bias in this study found that 32.862% of the variance, which is less than the 50%. In addition, Kock [51] stated that if all VIFs resulting from a comprehensive collinearity test are equal to or less than 5, the model can be considered free of common method bias; hence, no common method bias was discovered in this study. Organisational leadership capability (1.600), learning orientation (1.983), strategic flexibility (1.491), ambidextrous capability (1.775), organisational performance (1.226), All structural components of this model were evaluated lower than 3.3, showing absence of CMB.

### 3.4 Multivariate normality

This study examined multivariate normality using the Web Power web tool (source: https://webpower.psychstat.org/wiki/tools/index). Web Power computed the multivariate skewness and kurtosis coefficients for Mardia. In consequence, the *p*-value was 0.000 less than 0.05, confirming the existence of multivariate non-normality.

### 3.5 Data analysis method

PLS-SEM is a causal modelling technique that maximizes the variance explained of latent components [52]. PLS-SEM was utilized because to the exploratory and non-normal nature of this study. According to the methods proposed by Hair et al. [52] the analysis was reported that the methodologies include indicator reliability, internal consistency reliability, convergent validity, discriminant validity, average variance extracted (AVE), effect size, path coefficient estimations, and predictive significance.

## 4 Findings

### 4.1 Demographic characteristics

The characteristics for 524 respondents were based on age, gender, education, monthly income, and length of occupation, as presented in Table 1. Majority of the respondents were male with 78.6%, 53.6% of the respondents were doctoral or professor level and 46.4% were Master degree graduates. and 21.4 were female. 29.2% of them were under 30 years old to 40 years old, 25.2% were 41 years old to 50 years old, and 23.3% were 51 years old to 60 years old, and 22.3% were above 60 years old. In addition, 44.3% were earned between Rp. 2.500. 000 to Rp. 5.000.000, 33.8% were earned between Rp. 5.000.001 to Rp. 10.000.000, 16.8% were earned between Rp. 10.000.001 to Rp. 15.000.000, and 5.2% were earned above Rp. 15.000.000. based on length of occupation, most of the respondents worked between 6 and 10 years (38.9%), 26.5% worked between 11 and 15 years, 29.2% worked between 15 and 20 years old, and 5.2% worked more than 20 years.

**Table 1:** Respondents characteristics.

	*N*	*%*		*n*	*%*
*Gender*			*Education*		
Male	412	78.6	Master degree	243	46.4
Female	112	21.4	Doctoral/Professor level	281	53.6
Total	524	100	Total	524	100
*Age group*			*Monthly income*		
31–40 years	153	29.2	Rp. 2.500.000-Rp. 5.000.000	232	44.3
41–50 years	132	25.2	Rp. 5.000.001-Rp. 10.000.000	177	33.8
51–60 years	122	23.3	Rp. 10.000.001-Rp. 15.000.000	88	16.8
>60 years	117	22.3	above Rp. 15.000.000	27	5.2
Total	524	100	Total	524	100
*Length of Occupation*					
6–10 years	204	38.9			
11–15 years	139	26.5			
16–20 years	153	29.2			
>20 years	28	5.3			
Total	524	100			

### 4.2 Validity and reliability

The descriptive data and item dependability are presented in Table 2. The mean and standard deviation of the variables (organisational leadership capability, learning orientation, strategic flexibility, ambidextrous capability and organisational performance) are presented. As a conservative metric of internal consistency reliability, Cronbach's alpha is utilized. All variables had Cronbach's alpha values better than 0.75. This demonstrates that every item is trustworthy. According to Hair et al. [52], composite reliability is also applicable. 0.7 is the minimal value required to achieve composite dependability. The composite reliability values for all variables are greater than 0.80, as indicated in Table 2. In addition, the Dijkstra–Hensele's rho values for each variable exceed 0.70. In order to attain convergent validity, the AVE must be greater than 0.50. All AVE values are greater than 0.50, indicating acceptable convergent validity in this study. In addition, the variance inflation factors (VIFs) were examined to determine multicollinearity. The VIF values for each variable are less than 3.3 showed that there was no significant multicollinearity concern.

**Table 2:** Validity and reliability.

Variables	Items	Mean	Standard Deviation	Cronbach's Alpha	Dijkstra-Hensele's *rho*	Composite Reliability	Average Variance Extracted	Variance Inflation Factors
OLC	4	4.52	0.554	0.713	0.719	0.820	0.534	1.595
LO	5	4.23	0.586	0.788	0.796	0.855	0.542	1.878
SF	5	4.38	0.513	0.787	0.800	0.852	0.536	1.457
AC	4	4.30	0.573	0.786	0.803	0.860	0.607	1.764
OP	3	4.35	0.598	0.809	0.834	0.886	0.721	–

**Note:** OLC: Organizational Leadership Capability; LO: Learning Orientation; SF: Strategic Flexibility; AC: Ambidextrous Capability; OP: Organizational Performance.

**Fig. 2:**
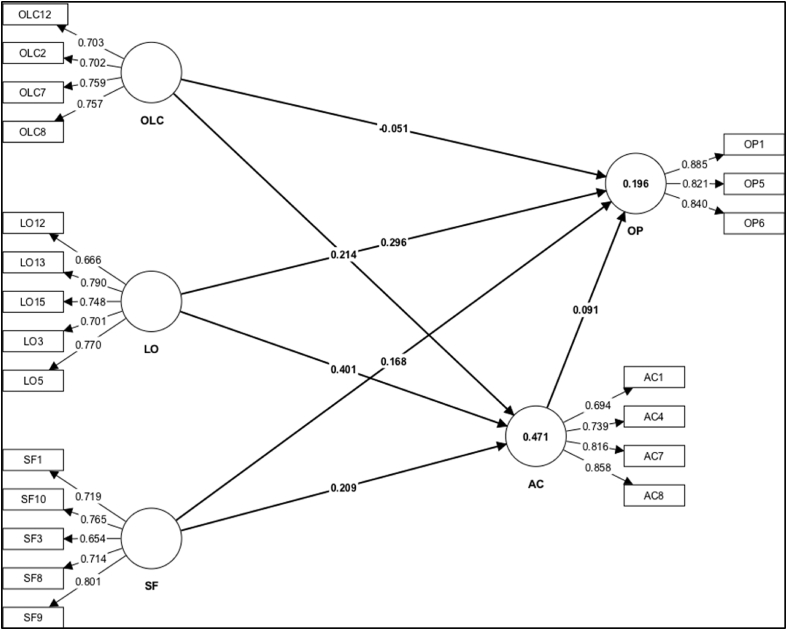
Measurement model and findings.

**Fig. 3:**
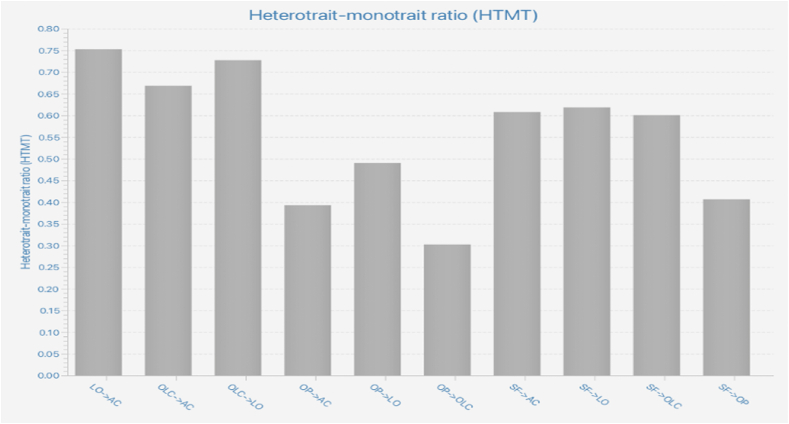
Heterotrait–Monotrait ratio.

All loading values (as presented in Fig. 2) are more than 0.5 and higher than the respective cross-loading values, confirms the discriminant validity. Fornell and Lacker criterion and Heterotrait–Monotrait ratio were utilized to test the discriminant validity of the components in this study. The Fornell–Lacker criterion (see Table 3) is applied to evaluate the discriminant validity by comparing the square root of AVE retrieved from each component to the correlation between constructs [52]. Based on Heterotrait–Monotrait ratio (HTMT) method estimates the discriminant validity of the construct on Fig. 3 displays the findings of the Fornell–Lacker and HTMT analyses, which found strong relationships between the components.

**Table 3:** Fornell-Larcker criterion.

	AC	LO	OLC	OP	SF
AC	0.779				
LO	0.624	0.736			
OLC	0.533	0.551	0.730		
OP	0.335	0.409	0.240	0.849	
SF	0.511	0.502	0.468	0.339	0.732

**Note:** OLC: Organizational Leadership Capability; LO: Learning Orientation; SF: Strategic Flexibility; AC: Ambidextrous Capability; OP: Organizational Performance.

### 4.3 Path analysis

As presented in Table 4, the findings revealed that the relationship between organizational leadership capability towards ambidextrous capability (H1a). This finding indicated that organizational leadership capability had a positive and significant effect towards ambidextrous capability (*β* = 0.241; *p*-value = 0.000). However, the correlation between organizational leadership capability and organisational performance had no significant effect towards (H1b) (*β* = −0.051; *p*-value = 0.229). The relationship between learning orientation on ambidextrous capability (H2a) was indicated a positive and significant effect (*β* = 0.401; *p*-value = 0.000). This result indicated that learning orientation had a positive and significant effect towards ambidextrous capability. Next, the finding of this study showed that learning orientation had significant effect towards organizational performance (H2b) (*β* = 0.296; *p*-value = 0.000). The relationship between strategic flexibility and ambidextrous capability had significant link (H3a) which showed by (*β* = 0.209; *p*-value = 0.001). In addition, the significant relationship was found between strategic flexibility and organisational performance in this study (H3b) (*β* = 0.168; *p*-value = 0.016). In contrast, the result of this study also confirmed that (H4) ambidextrous capability had no significant correlation towards organisational performance (*β* = 0.019; *p*-value = 0.129).

The *r*^*2*^ value of 0.471 indicates that around 47% of the variation in AC can be explained by OLC, LO, and SF. The *f*^*2*^ values indicate that the effect of OLC (0.056), LO (0.188), and SF (0.057) on AC is relatively low. The *r*^*2*^ value of 0.196 showed that around 2% of the variation in OP can be explained by OLC, LO, SF and AC. The *f*^*2*^ values indicate that the effect of OLC (0.005) is relatively low, whereas the effect of OLC (0.002), LO (0.057), and SF (0.023) on OP is relatively low. Finally, the *r*^*2*^ value of 0.471 indicates that around 47% of the variation in the OP can be explained by AC. The *f*^*2*^ value of 0.005 indicates a small effect of AC on the OP amongst the private high schools in Indonesia.

### 4.4 Mediating effects

Table 4 also presented the mediating effect of ambidextrous capability. The finding showed that ambidextrous capability was unable play mediating variable to influence the antecedences and consequence in this study which showed by *p*-value each antecedence was higher than 0.05. Consequently, ambidextrous capability had no mediating role in this study (hypotheses 5 was rejected).

**Table 4:** Path analysis.

Hypo		Beta	*t*	*p*	*r*^2^	*f*^*2*^	Decision
*Factors Ambidextrous Capability*							
H_1a_	OLC → AC	0.214	3.784	0.000		0.056	Supported
H_2a_	LO → AC	0.401	6.120	0.000	0.471	0.188	Supported
H_3a_	SF → AC	0.209	3.138	0.001		0.057	Supported
*Factor affecting Organizational Performance*							
*H4*	AC → OP	0.091	1.130	0.129		0.005	Rejected
*H5a*	OLC → OP	−0.051	0.743	0.229	0.196	0.002	Rejected
*H5b*	LO → OP	0.296	4.433	0.000		0.057	Supported
H5c	SF → OP	0.168	2.143	0.016		0.023	Supported
*Mediating effect of Ambidextrous Capability*							
H5b	OLC → AC → OP	0.020	1.057	0.145			Rejected
H5b	LO → AC → OP	0.037	1.098	0.136			Rejected
H5c	SF → AC → OP	0.019	0.981	0.163			Rejected

**Note:** OLC: Organizational Leadership Capability; LO: Learning Orientation; SF: Strategic Flexibility; AC: Ambidextrous Capability; OP: Organizational Performance. **Source:** Author (s) own compilation.

### 4.5 Analysis of the importance–performance matrix (IPMA)

**Fig. 4:**
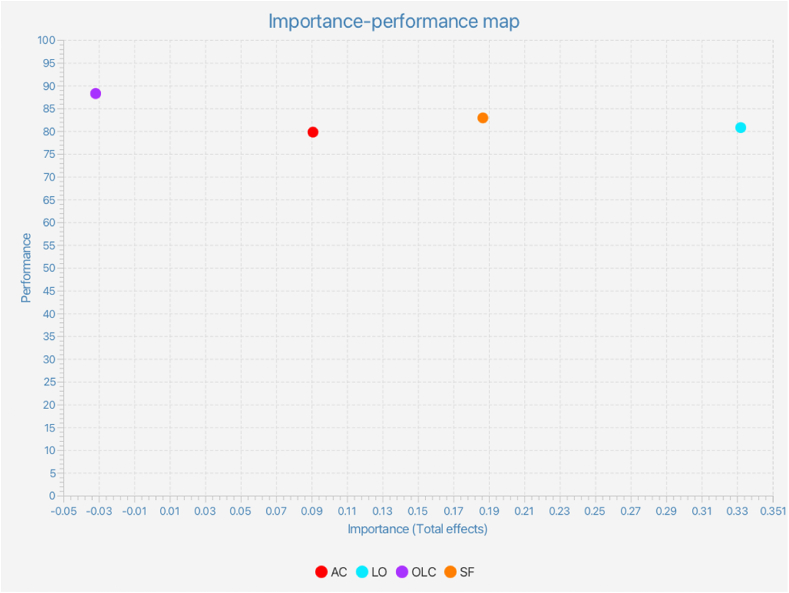
Importance–performance matrix.

The importance–performance matrix analysis (IPMA) extends the results of PLS-SEM by incorporating each construct's performance. As a result, conclusions may be reached on both importance and performance, which is specifically accomplished via PLS to assess the robustness of the study results by taking the performance of each component action into consideration. The findings (as seen in Fig. 4) indicate that the learning orientation has the greatest impact on organizational performance, followed by strategic flexibility, ambidextrous capability, and organisational leadership capability.

## 5 Discussion

Strategy diversification and integration enable leaders of private HEIs to identify opportunities and exploit capabilities. Recognising and exploiting external resources and reconfigure technological changes and knowledge are needed to transform into new capabilities for higher organisational performance [22]. The purpose of this study was to evaluate the mediating effect of ambidextrous capability on the influence of organisational leadership capability, learning orientation, and strategic flexibility on the performance of HEIs in Indonesia. This study proposed the dynamic capability theory to support the findings and explain the correlations among the constructs.

This study confirmed the significant and positive influence of organisational leadership capability on ambidextrous capability. Kivipold [30] presented similar results on the influence of organisational leadership capability, which was expressed as the interaction between the main behavioural principles of an organisation. Our findings indicated that the dynamic capability strongly contributes to the capabilities of organisational leaders (rectors and deans), as the decision-makers, to adopt ambidextrous capability for organisational success. Furthermore, the current study found no significant correlation between organisational leadership capability and the performance of private HEIs in Indonesia, which contradicted the results presented by Siswanto et al. [53]. The prior study demonstrated the significant influence of organisational leadership capability on performance and innovation. The study further argued that the simultaneous exploration and exploitation activities are beneficial in improving the sustainable performance of HEIs. Organisational leadership capabilities alone could not improve organisational performance without the intensification of new capabilities in any rapidly progressing business environment [54,55]. Moreover, dynamic capability improvement is required for leaders of HEIs to change strategies and improve the performance of HEIs [56]. However, the current study's findings on the insignificant direct relationship between organisational leadership capability and the performance of HEIs suggest the need for leaders of private HEIs to increase their capabilities and competency levels regarding the advantages of knowledge enhancement and strategic learning implementation in HEIs.

The current study also discovered the significant influence of learning orientation on ambidextrous capability. Liao and Barnes [36] reported similar findings on how learning orientation can inspire new ideas and knowledge to attain innovation. Organisational learning focuses on the use of existing knowledge and the acquisition of new knowledge to create organisational strategy. Adding to that, the current study confirmed the positive relationship between organisational learning and the performance of private HEIs. Siswanto et al. [53] reported similar findings on how creativity generates ideas to carry out activities and develops new ideas into opportunities. The present study highlighted that the importance of learning orientation for the organisational performance through gaining capabilities can provide superior outcomes, such as curriculum development. Considering that, the performance of private HEIs in Indonesia clearly requires appropriate leadership skills, capabilities, and efforts to comprehend strategic planning, challenges, opportunities, and technologies, as well as to gain new knowledge, such as certification, seminar, and training as part of human resource investment.

In addition, the current study showed the significant influence of strategic flexibility on ambidextrous capability. Similarly, Singh et al. [57] highlighted the importance of organisational capability in creating strategic flexibility by adjusting the external environment, such as new technology, consumers' need, and market fluctuation. Besides that, the current study found the significant relationship between strategic flexibility and organisational performance. The obtained results described that strategy formulation enables leaders to achieve superior organisational performance. This may be attributed to the fact that the performance of private HEIs must adapt to the rapid changes in curriculum quality and learning methods according to the students’ needs. A more flexible learning strategy would result in better organisational performance. However, the current study found no relationship between ambidextrous capability and the performance of private HEIs. The findings of the current study were not in line with the findings of prior studies [58]. These studies pointed out that ambidextrous capability can reduce the length of time needed to gain sustainable competitive advantage. Such capability enables a leader to transfer knowledge that involves exploration and exploitation in order to achieve long-term organisational performance [58]. The current study suggested the need to adopt new learning platforms in the education industry, such as Webex meeting, Zoom meeting, and Google Classroom, to enhance the performance of private HEIs in Indonesia—these platforms are vital in improving the performance of these institutions. The capabilities of leaders to gain and use new knowledge to establish a new strategic programme enable private HEIs to achieve outstanding organisational performance.

Based on the obtained results, the current study confirmed the insignificant mediating effect of ambidextrous capability on the relationships of organisational leadership capability, learning orientation, and strategic flexibility with the performance of HEIs, which contradicted the findings reported by Huang and Li [44] and Severgnini et al. [43]. These prior studies concluded that ambidextrous capability enable organisations to be proficient in the exploitation and exploration of new knowledge and to anticipate consumers' preferences and technological advances for innovation. However, the role of ambidextrous capability was found to be not critical for private HEIs in Indonesia. In other words, leaders of HEI do not improve their ambidextrous capability and strategies in terms of curriculum development, learning system, and improvement of lecturers' knowledge and strategic flexibility to promote superior organisational performance. Moreover, Severgnini et al. [43] argued that ambidextrous organisations should show high levels of both exploitative and explorative strengths in building and maintaining strategic decision-making and monitoring the organisational strategy towards achieving organisational performance. After all, ambidextrous capability is a process with high degrees of both exploitative and explorative strengths to establish and maintain strategic decision-making and monitoring the organisational strategy to achieve organisational success. Therefore, rectors and deans of HEIs should participate and contribute to the development of new ideas and their incorporation into the curriculum and the students’ creativity. It is strongly recommended that the capabilities of private HEIs to develop and intensify new techniques for the management of educational lesson implementation are improved, as changes require extensive outreach.

In view of the above, the obtained results of this study highlighted no mediating role of ambidextrous capability among the private HEIs in Indonesia. The inconsistencies may be due the inability to capture opportunity identification and exploitation capabilities among these institutions in Indonesia. The process of identifying opportunities is strongly tied to the acquisition of knowledge. After uncovering a new opportunity, it is necessary to integrate new knowledge with existing knowledge stocks, processes, products, or strategies in order to capitalise on this opportunity. The management systems, organisational structures, values, and cultures within the domain of higher education may also influence the incorporation of new information. As a result, this study was not able to demonstrate the mediating effect of ambidextrous capability on organisational performance. Therefore, additional policy and strategy are needed to improve the competitive advantage among private HEIs in Indonesia.

## 6 Conclusion

The main purpose of this study was to evaluate the mediating effect of ambidextrous capability on the relationships of organisational leadership capability, learning orientation, and strategic flexibility with the performance of private HEIs in Indonesia. This study demonstrated no significant effect of ambidextrous capabilities on organisational performance. Based on the obtained results, the dynamic capability theory was deemed inadequate to elucidate the organisational performance of selected private HEIs in Indonesia. The results of this study highlighted the need to explore the model constructs under the dynamic capability theory, specifically on how ambidextrous capability can help HEIs to enhance their organisational performance.

From the viewpoints of 524 rectors and deans of private HEIs, the institutions were reported to possess inadequate preparation to embrace or employ innovative teaching systems and curriculum for teaching or research. This is exemplified by the lack of engagement with learning strategies and capabilities among the private HEIs in Indonesia, which may be primarily due to the difficulty and lack of awareness among leaders to learn new strategies and learning techniques. In fact, compatibility and observability concerns have been identified as impediments to the implementation of the global teaching curriculum.

Lastly, this study proposed a number of recommendations that may benefit decision-makers and related practitioners, particularly in private HEIs, to address the aforementioned challenges and improve organisational performance. Individually, private HEIs should consider investing in developing, disseminating, and adopting new curriculum and teaching methods for their research and seminars. It is also necessary for private HEIs to make greater efforts to overcome traditional and negative perspectives of global curriculum, new knowledge, and system approaches. At the community level, these institutions are suggested to encourage their peers by including more adoption strategies in their curriculum and enhancing the lecturers’ adoption capacities.

This study also revealed that the majority of recommendations have been made at the institutional level. Private HEIs must provide institutions, lecturers, and students with more awareness conferences and training programmes in order to master the creation, implementation, and adoption of new system learning and teaching processes. Indeed, rewards and recognition from the respective institutions are necessary for the rectors or deans to increase interest in this area.

In conclusion, the phenomenon of organisational performance remains a challenge considering that the cooperation between institutions and all individuals involved in the institutions requires a high degree of openness to the academic and educational communities in general. Apart from the existing constructs, there may be other constructs or factors that can justify the influence of dynamic capability on the performance of HEIs.

### 6.1 Theoretical implication

The dynamic capability theory was employed to explain the conceptual model in this study. In the presence of uncertainty, the functions of dynamic capability become more crucial to execute the decision of the organisation regarding what and how to apply strategies [59]. The functions of dynamic capability are sensing (identification and assessment of threats, opportunities, and potential users’ need), seizing (mobilisation of resources to address fresh opportunities to gain value), and transforming to help the organisation explain the environmental uncertainty and transform this situation to become an advantage. This study developed a conceptual model to examine the mediating effect of ambidextrous capability on the relationship between organisational leadership capability, learning orientation, strategic flexibility, and the organisational performance of private higher education institutions. A throughout understanding of how crucial role of dynamic capability potentially grows and provides a better organisational performance in a hypercompetitive market [22].

### 6.2 Practical implication

The combination of internal capabilities (financial, resources, and value) and external capabilities (technological changes, knowledge, and market opportunities) must be maximally used to increase the capabilities of higher education institutions to acquire sustainable performance [18]. Apart from that, this study revealed that the rector's capability to exploit internal capabilities is still less. Human competency, the diversification of the curriculum, planning strategy, benchmark and R&D investment should be improved in developing countries. The capability to convert a threat into an opportunity is also an important factor to obtain higher education success in the future. Furthermore, rectors and deans of private HEIs should utilise the information and new issues to update the strategic formulation as well as technological resources can transform weaknesses into strengths to achieve organizational performance.

The ambidextrous diversification delivers sustainable competitive advantage and strengthens diversification to encounter unpredictable environments for the performance of higher education institutions. Ambidextrous capability is essential factor which offers and enhance the quality of the curriculum and teaching to achieve better performance. The process of acquiring, deploying, integrating, and reconfiguring resources and capabilities are required as strategic flexibility toward new curriculum. Finally, this study has shown that ambidextrous diversification of curriculums, the development of human capabilities, and the intention to learn can achieve sustainable performance.

### 6.3 Limitation

This study examined the mediating effect of ambidextrous capability on organisational performance but found inadequate evidence to support the dynamic capability theory (DCT). In this sense, based on the theory of dynamic capacity (TDC) and the findings of this study, this study presented several recommendations for future research to address the limitations of this study. Firstly, this study sampled a total of 524 rectors and deans of private HEIs in Indonesia. This study found no significant effect of organisational leadership capability and dynamic capability on organisational performance among the selected private HEIs in this low-income nation. Thus, it is recommended for future research to expand the scope for better insights on these relationships by exploring potential factors that influence the long-term performance of HEIs in middle-income and developed-income nations. Moreover, this study found the lack of strategic planning, knowledge, and ambidextrous capability in developing lecturers’ capabilities in teaching and learning strategies among the leaders of private HEIs in Indonesia. It is necessary to consider different organisational environments in order to obtain more comprehensive findings on the dynamic capability theory and ambidextrous capability at higher accuracy. Finally, this study adopted the cross-sectional research design, which focused on a specific period of time. Therefore, it is recommended for future research to consider longitudinal data to obtain better understanding on how to enhance the organisational performance.

## Author contribution statement

Abdullah Al Mamun: Conceived and designed the experiments; Analyzed and interpreted the data; Wrote the paper.

Marvello Yang; Anas A. Salameh: Conceived and designed the experiments; Contributed reagents, materials, analysis tools or data; Wrote the paper.

## Funding statement

This study was supported by Prince Satam bin Abdulaziz University (PSAU/2023/R/1444).

## Data availability statement

Data included in article/supp. material/referenced in article.

## Appendix A Supplementary data

Supplementary Table 1. Survey Instrument Supplementary Table 1. Survey Instrument
