# Supplementary material for: Leadership, capability and performance: A study among private higher education institutions in Indonesia
Source: Heliyon. 2023 Jan 18;9(1):e13026. doi: 10.1016/j.heliyon.2023.e13026 (PMC9873678; doi:10.1016/j.heliyon.2023.e13026)
Supplement: Supplementary Table 1. Survey Instrument [file mmc1.docx]

**Supplementary Table 1.** Survey Instrument

| Codes | Questions |
| --- | --- |
| OLC-Item 2 | I have set the main purpose objectives of my higher education. |
| OLC-Item 7 | I arrange gatherings that are not work-related to obtain intimate among lecturers. |
| OLC-Item 8 | I arrange work-related gatherings to discuss next teaching planning. |
| OLC-Item 12 | We make important decisions using the process of discussion with lecturers. |
| LO-Item 3 | The sense around here is that lecturers learning is an investment, not an expense. |
| LO- Item 5 | Learning orientation has given lecturers a better intellectual capability for future challenges. |
| LO-Item 12 | Our higher education always monitors any changes in education market. |
| LO-Item 13 | To gain a better performance, our higher education conducts the benchmark in some reputable higher educations. |
| LO-Item 15 | I always implement continuous improvement of strategies through any programs that I have made. |
| SF-Item 1 | Our higher education always forms new institution structures to obtain new ideas and strategies from different people during five years. |
| SF-Item 3 | Our higher education supports lecturers’ performance such as research and teaching. |
| SF-Item 8 | The curriculum that is made always follows changes in the current competitive market in domestic as well as global education institution. |
| SF-Item 9 | Our higher education makes new innovation in our teaching methods in the class to achieve a better performance. |
| SF-Item 10 | Our higher education adopts faster change in using digital platform in learning process. |
| AC-Item 1 | Our higher education has capability to change the quality of our curriculum. |
| AC-Item 4 | We frequently utilize new opportunities in new markets to form our strategy. |
| AC-Item 7 | We increase economies of scales to enhance the efficiency of operational budgets. |
| AC-Item 8 | Our higher education expands strategy in our curriculum and lecturers’ capabilities for competing with other higher educations in Indonesia. |
| OP-Item 1 | Our higher education uses the full abilities and potential of its lecturers. |
| OP-Item 5 | Our higher education successfully implemented strategy such as developing lecturers’ capability, designing new curriculum and students’ development and creativity. |
| OP-Item 6 | Our higher education successfully withstands competition in the future. |

**Note:** OLC: Organisational Leadership Capability; LO: Learning Orientation; SF: Strategic Flexibility; AC: Ambidextrous Capability; OP: Organisational Performance
